# Significance of Size of Lymph Node Metastasis on Postsurgical Stimulated Thyroglobulin Levels After Prophylactic Unilateral Central Neck Dissection in Papillary Thyroid Carcinoma

**DOI:** 10.1245/s10434-012-2385-4

**Published:** 2012-05-08

**Authors:** Brian Hung-Hin Lang, Alex H. Tang, Kai Pun Wong, Tony W. Shek, Koon Yat Wan, Chung-Yau Lo

**Affiliations:** 1 Division of Endocrine Surgery, Department of Surgery, The University of Hong Kong, Hong Kong SAR, China; 2 Department of Anatomical Pathology, Queen Mary Hospital, Hong Kong SAR, China; 3 Department of Clinical Oncology, The University of Hong Kong, Hong Kong SAR, China

## Abstract

**Background:**

The prognostic significance of size of central lymph node metastasis (CLNM) in papillary thyroid carcinoma (PTC) remains unknown. Because postsurgical detectable stimulated thyroglobulin (DsTg) after radioiodine ablation may imply persistent or recurrent disease, we evaluated the association between size of CLNM and rate of DsTg in patients with PTC who underwent unilateral prophylactic central neck dissection.

**Methods:**

To be eligible for analysis, the prophylactic central neck dissection specimen with <3 central lymph nodes (CLNs) or size of CLNM ≥1 cm as measured under the microscope was excluded. Of 132 specimens, 89 (67.4 %) were eligible. Forty patients (44.9 %) had no metastasis or pN0, 20 (22.5 %) had micrometastasis (<2 mm) or pN1mic and 29 (32.6 %) had macrometastasis (≥2 mm) or pN1mac. Postablation sTg level was measured 9 months after surgery. A multivariable analysis was conducted to identify independent factors for postablation DsTg.

**Results:**

Larger-sized CLNM correlated significantly with younger age (*p* = 0.028), greater number of CLN retrieved (*p* = 0.016), greater number of metastatic CLN excised (*p* < 0.001), higher metastatic CLN ratio (*p* = 0.006) and postablation sTg level (*p* = 0.012). In the multivariable analysis, after adjusting for tumor size and metastatic CLN ratio, size of CLNM was an independent predictor of postablation DsTg (odds ratio 1.56, 95 % confidence interval 1.09–2.24, *p* = 0.015). Relative to pN0, the odds ratios for postablation DsTg in pN1mic and pN1mac were 2.53 (95 % confidence interval 0.35–19.00, *p* = 0.351) and 5.81 (95 % confidence interval 1.22–27.70, *p* = 0.027), respectively.

**Conclusions:**

Size of CLNM was an independent factor for DsTg 9 months after surgery. Patients with pN1mac were almost 6 times more likely to have postablation DsTg than those with pN0 or pN1mic.

Papillary thyroid carcinoma (PTC) is the most common type of differentiated thyroid carcinoma, and its age-adjusted incidence has doubled in the last 25 years.^1^ Despite its relatively good prognosis, with a cancer-specific survival above 90 %, locoregional recurrence (LRR) is common.^2^ With recognition of the stepwise progression of lymph node metastasis (LNM) originating from the central (level VI) to the lateral compartment (levels II–V), a growing number of surgeons are advocating routine prophylactic central neck dissection (pCND) at the time of the total thyroidectomy.^3^ Although the role of pCND has remained controversial because there is little evidence to suggest that it improves long-term outcomes when compared to no pCND, analysis of short-term markers (such as stimulated thyroglobulin) (sTg) seems to suggest pCND may improve short-term outcomes.^4^–^7^

Because patients with clinically apparent or palpable LNM in the lateral compartment (or N1b) are at significantly higher risk of developing LRR and distant metastases when compared to those with clinically inapparent LNM, size of LNM is believed to be a prognostic factor in patients with PTC.^8^–^14^ However, to our knowledge, no study has specifically addressed the significance of size of central LNM (CLNM) in patients who routinely undergo pCND. For patients who undergo pCND after total thyroidectomy, the incidence of histologically proven CLNM ranged 40–50 %.^4^–^7^ However, because these CLNM are often small in size and may only be seen under high-power magnification, their clinical significance have been questioned.^12^,^13^ A recent study reported a series of 72 patients who underwent a pCND and found that none had CLNM ≥2 mm in size (macrometastasis or pN1mac), whereas only 18 patients (25.0 %) had CLNM <2 mm (micrometastasis or pN1mic) present in the central lymph nodes (CLNs).^15^ We hypothesized that size of CLNM might have a negative impact on prognosis, with larger CLNM leading to poorer prognosis. Postsurgical detectable sTg level (DsTg) after radioiodine (RAI) ablation is a good surrogate for persistent or recurrent disease after total thyroidectomy.^16^–^19^ Therefore, the present study aimed to evaluate the association between size of CLNM and rate of postablation DsTg in patients who underwent an unilateral pCND with particular emphasis on the incidence and significance of pN1mic and pN1mac.

## Patients and Methods

From 2005 to 2011, a total of 250 consecutive patients with PTC underwent surgery in our institution. All were managed by the same surgical team. Of these, 142 (56.8 %) underwent a routine unilateral pCND at the time of the total thyroidectomy. None had evidence of CLNM preoperatively on ultrasonography or intraoperatively. Patients with concomitant lateral lymph node metastases (N1b) or distant metastases (M1) were excluded. All operations were performed by two endocrine surgeons. The decision for a prophylactic unilateral central neck dissection (pCND) was based on the personal preference of the operating surgeon and not based on tumor size or other tumor characteristics. However, there was a tendency to perform more pCND in the latter part of the study period. During the study period, all resected specimens were examined by the same group of pathologists in our institution by a standardized technique. Specimens containing <3 CLNs retrieved during pCND (*n* = 38) were excluded to reduce the possibility of nodal under-staging.^20^ Also, patients with anti-thyroglobulin (Tg) autoantibody titer of >400 (*n* = 10) were excluded. Therefore, a total of 94 patients were eligible for analysis. Of these, 40 (42.6 %) had no demonstrable LNM on hematoxylin and eosin stain (or pN0) (group I), whereas 54 (57.4 %) had ≥1 demonstrable metastatic focus in CLN on hematoxylin and eosin stain. To measure the size of the CLNM, the relevant slides were retrieved and reexamined by two independent pathologists (A.H.T., T.W.S.). All metastatic foci were measured to the nearest tenth of a millimeter with a built-in computerized measurement program on the microscope (Nikon Eclipse E600). For patients with multiple metastatic foci, only the largest microscopic focus was recorded. To further ensure that the study only evaluated the significance of size of CLNM in the prophylactic setting, 5 patients with CLNM ≥1 cm were excluded because they could not be considered as prophylactic. To evaluate the association between size of CLNM and other clinicopathologic characteristics, patients were categorized into 3 groups: group I (pN0) (*n* = 40), group II (size of CLNM <2 mm) (pN1mic) (*n* = 20) and group III (size of CLN metastasis ≥2 mm) (pN1mac) (*n* = 29).

## Methods

All relevant clinical, laboratory, radiologic and perioperative data were collected prospectively, and follow-up data were regularly updated in a computerized database. The present study protocol was approved by the local institutional review board. Patient clinicopathologic features, sTg and postoperative outcomes were compared between the three groups.

### Management of PTC

Details of surgical treatment, criteria for RAI ablation, postoperative care and follow-up protocol have been described previously.^11^ In brief, total thyroidectomy was the preferred procedure for all patients with a preoperative diagnosis of PTC. A similar extent of unilateral pCND was performed for all patients, regardless of tumor size or extent.^6^ The pCND consisted of the removal of all nodes and fibro-fatty tissue extending vertically from the hyoid bone to the thoracic inlet and laterally from the medial border of common carotid artery to the midline of the trachea. The ipsilateral recurrent laryngeal nerve was mobilized and skeletonized along its entire cervical course. Parathyroid autotransplantation was readily performed. sTg was defined as a Tg level measured in the presence of thyroid-stimulating hormone (TSH) >30 mIU/L either by 4-week thyroxine withdrawal or recombinant TSH injections. The preablation sTg levels were taken approximately 2 months after surgery (at the time of RAI ablation), while the postablation levels were taken approximately 9 months after surgery (6–7 months after RAI ablation or at the time of the whole body scan). Tg autoantibodies were measured at the same time. The decision for RAI ablation was based on the presence of at least one or more risk factors such as tumor size >1 cm, LNM, age older than 45 years, extrathyroidal extension, macroscopic postoperative residual disease in the neck and distant metastasis, and were not dependent on the preablative sTg level. Three giga becquerels or 80 mCi I^131^ was the standard dose. TSH suppression to <0.1 μg/L was recommended for high- and intermediate-risk patients.

### Follow-up Protocol

All postsurgical patients were followed up within 4 weeks in a specialized combined oncology clinic. A follow-up visit was conducted at 3-month intervals in the first 2 years, every 6 months in the subsequent 3 years, and annually thereafter. Clinical examination, neck ultrasonography and nonstimulated Tg level were done during follow-up visits. LRR was confirmed by fine-needle aspiration cytology or histology.

### Laboratory Methods

All postoperative sTg levels were measured at the same laboratory by the same immunometric assay. The assay used was Immulite 2000 (Diagnostic Products, Roche, Los Angeles, CA). This was calibrated against the CRM-457 standard. A sTg level of ≥0.5 μg/L was considered a detectable stimulated thyroglobulin (DsTg). Normal reference range was <0.5–55 μg/L, and sensitivity was <0.2 μg/L).

### Statistical Analyses

**Fig. 1:**
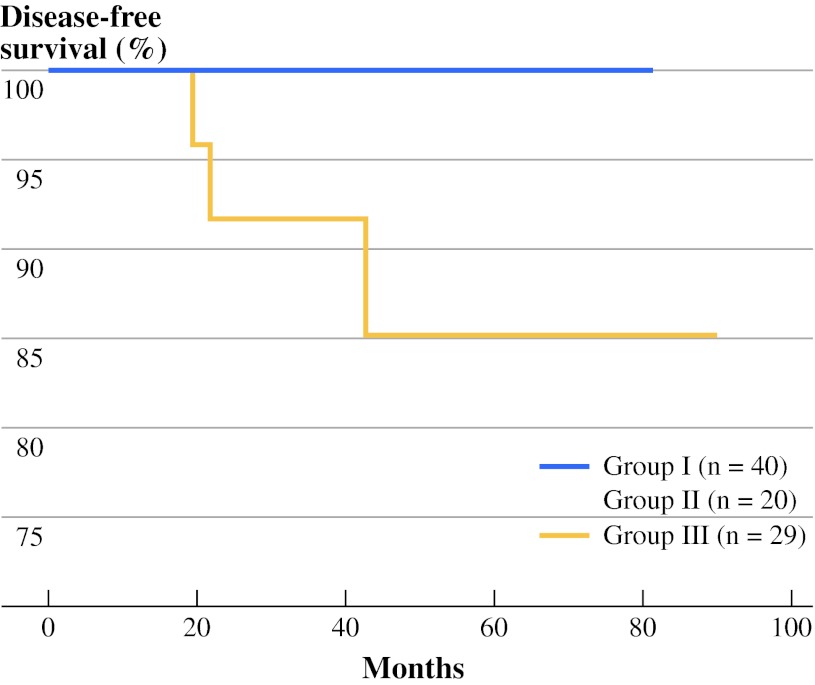
Cumulative disease-free survival curves of papillary thyroid carcinoma between those with no central lymph node (CLN) metastasis (pN0) (group I), with CLN micrometastases (pN1mic) (group II), and with CLN macrometastases (pN1mac) (group III)

Statistical analyses was performed by χ^2^ or Fisher’s exact test to compare categorical variables, and Mann–Whitney *U* or Kruskal–Wallis test was used to compare continuous variables between groups. To evaluate the correlation between two continuous variables, the Spearman rank correlation test was performed. Continuous variables were expressed as medians with ranges. Variables that were significant in the univariate analysis were entered into multivariate analysis. Binary logistic regression analysis with a variable entrance criterion of 0.05 or less was conducted to identify factors associated with postablative DsTg after surgery. Disease-free survivals were estimated by the Kaplan–Meier method and compared with the log-rank test (Fig. 1). All statistical analyses were performed by SPSS software, version 18.0 (SPSS, Chicago, IL).

## Results

In our cohort, most were women (75.3 %) and ethnic Chinese (89.9 %). The median age at operation was 48.0 (range 8.1–81.3) years, and the median follow-up period was 29.1 (range 9.3–90.1) months. The median primary tumor size was 1.5(range 0.3–6.0) cm, with 8 (9.0 %) being microcarcinomas (<1 cm). The median number of metastatic CLNs was 1 (range 1–18), and the median number of CLNs collected was 6 (range 3–21). The median size of CLNM was 2.6 (range 0.1–6.9) mm.

Table 1 shows a comparison of patient clinicopathologic features, tumor, node, metastasis staging system (TNM) tumor stages, CLN ratio, and metastases, age, completeness of surgery, invasion, and size (MACIS) score between the three groups. The median age at operation was significantly different between the three groups (*p* = 0.003), with group III having the youngest median age of 43.0 years. There was an inverse correlation between age and size of CLNM (ρ = −0.315, *p* = 0.028). Gender, clinical presentation and tumor characteristics such as tumor size, multifocality, capsular invasion, extrathyroidal extension and coexisting thyroiditis were not significantly different between the three groups. Stage of PTC by TNM, MACIS score, postoperative complications and anti-Tg autoantibody titer were also similar between the three groups. Group III had significantly higher number of CLNs retrieved (*p* = 0.007), metastatic CLNs excised (*p* < 0.001) and metastatic CLN ratio (*p* = 0.010) than the other two groups. There was a direct correlation between size of CLNM and number of CLNs retrieved (ρ = 0.343, *p* = 0.016), size of CLNM and number of metastatic CLNs excised (ρ = 0.541, *p* < 0.001) and size of CLNM and metastatic CLN ratio (ρ = 0.387, *p* = 0.006). Group III also tended to have higher proportion of patients receiving RAI ablation after surgery than groups I and II (96.6 vs. 80.0 and 90.0 %, respectively), but this was not statistically significant (*p* = 0.198). In the preablation period the median sTg was similar, whereas in the postablation period the median sTg level was significantly different between the three groups (*p* = 0.012). The proportion of DsTg in groups I and II was reduced from 60 to 17.5 and 55 to 15 %, respectively, after RAI ablation, whereas the proportion in group III was reduced from 82.8 to 62.1 %. At the time of analysis, all patients were alive. In groups I and II, no patient developed LRR, whereas in group III, 2 developed ipsilateral lateral recurrences and 1 developed bilateral lateral recurrence. These three recurrences developed at 19.4, 21.9 and 42.9 months after surgery. All three recurrences had postablation DsTg 9 months after surgery, and their median sTg level was 87.0 (range 9.7–103.0) μg/L. The cumulative disease-free survival in groups I, II and III were not significantly different (*p* = 0.151), and the 5-and 10-year disease-free survivals in group III were 85.1 and 85.1 %, respectively.

**Table 1:** A comparison of patient characteristics between group I, II and III

Characteristic	Group I (*n* = 40)	Group II (*n* = 20)	Group III (*n* = 29)	*p*-value
Age at operation (y)	52 (17–81)	48 (8–73)	43 (21–67)	**0.003**
Sex				0.814
Male	9 (22.5)	6 (30.0)	7 (24.1)	
Female	31 (77.5)	14 (70.0)	22 (75.9)	
Clinical presentation				0.310
Incidental	13 (32.5)	9 (45.0)	7 (24.1)	
Symptomatic	27 (67.5)	11 (55.0)	22 (75.9)	
Tumor characteristics				
Tumor size (cm)	1.5 (0.3–3.8)	1.5 (0.3–4.0)	2.0 (0.5–6.0)	0.266
Multifocality	15 (37.5)	6 (30.0)	10 (34.5)	0.901
Capsular invasion	10 (25.0)	6 (30.0)	5 (17.2)	0.509
Extrathyroidal extension	14 (56.0)	4 (20.0)	6 (20.7)	0.331
Coexisting thyroiditis	8 (20.0)	4 (20.0)	7 (24.1)	0.378
Stage of PTC by TNM				0.198
Stage I/II	20 (50.0)	6 (30.0)	16 (55.2)	
Stage III/IV	20 (50.0)	14 (70.0)	13 (44.8)	
Size of CLN metastasis (mm)	–	1.1 (0.1–1.8)	4.2 (2.0–6.9)	**<0.001**
No. of CLNs retrieved	4 (3–18)	5.5 (3–9)	8 (3–21)	**0.007**
No. of metastatic CLNs excised	–	1 (1–6)	4 (1–18)	**<0.001**
Metastatic CLN ratio (%)^a^	–	29.2 (12.5–100)	69.2 (12.5–100)	**0.010**
MACIS score	5.2 (3.2–8.0)	4.9 (3.3–12.0)	4.3 (3.2–8.9)	0.094
Postoperative hypocalcemia				0.739
Temporary	5 (12.5)	4 (20.0)	6 (20.7)	
Permanent	1 (2.5)	0 (0.0)	1 (3.4)	
Postoperative vocal cord palsy^b^				0.348
Temporary	0 (0.0)	1 (2.5)	2 (3.4)	
Permanent	0 (0.0)	1 (2.5)	2 (3.4)	
Radioiodine ablation	32 (80.0)	18 (90.0)	28 (96.6)	0.198
Anti-Tg antibody (titers)				0.399
≤99	33 (82.5)	15 (75.0)	26 (89.7)	
100–400	7 (17.5)	5 (25.0)	3 (10.3)	
Preablation period				
TSH level (mIU/L)	62 (34–339)	48 (35–226)	57 (37–250)	0.826
Stimulated Tg level (μg/L)	1.2 (<0.5–15.0)	0.9 (<0.5–114)	3.4 (<0.5–196)	0.141
No. of DsTg	24 (60.0)	11 (55.0)	24 (82.8)	0.070
Postablation period				
TSH level (mIU/L)	87 (65–253)	91 (86–146)	68 (39–229)	0.595
Stimulated Tg level (μg/L)	<0.5 (<0.5–4.7)	<0.5 (<0.5–9.2)	0.8 (<0.5–110)	**0.012**
No. of DsTg	7 (17.5)	3 (15.0)	18 (62.1)	**0.001**
Locoregional recurrence	0 (0.0)	0 (0.0)	3 (10.3)	0.157

Boldface signifies *p*-value <0.05 or statistically significant

Continuous variables are expressed as median (range); categorical variables are expressed as *n* (%)

*PTC* papillary thyroid carcinoma, *TNM* 6th edition of the tumor, node and metastasis staging system, *CLN* central lymph node, *MACIS* metastases, age, completeness of surgery, invasion, and size, *TSH* thyroid-stimulating hormone, *DsTg* detectable stimulated thyroglobulin (>0.5 μg/L)

^a^CLN ratio = (no. of metastatic CLNs/no. of CLNs retrieved) × 100

^b^Calculated on the basis of number of nerves at risk

Table 2 shows the univariate analysis of clinicopathologic factors for postablation DsTg (i.e., > 0.5 μg/mL). Age, gender, clinical presentation and most tumor characteristics such as multifocality, capsular invasion, extrathyroidal extension and thyroiditis were similar between those with postablation DsTg and with undetectable sTg. However, relative to those with undetectable sTg, those with postablation DsTg had larger median primary tumor size (2.0 vs. 1.5 cm, *p* = 0.018), greater number of metastatic CLNs (2 vs. 1, *p* = 0.013), higher CLN ratio (66.7 vs. 20.0 %, *p* = 0.003) and larger size of CLNM (4.8 vs. 1.6 mm, *p* < 0.001), but the proportion of patients with metastatic CLN (pN1a) was similar between the two groups (*p* = 0.114). When size of CLNM was categorized into types of metastasis (pN0, pN1mic and pN1mac), those with postablation DsTg had a significantly higher proportion of pN1mac (67.7 vs. 10.9 %) but a lower proportion of pN0 (20.6 vs. 60.0 %) and pN1mic (11.8 vs. 29.1 %) than those with undetectable sTg (*p* = 0.002). There was a significant direct correlation between the size of CLNM and postablation sTg level (ρ = 0.428, *p* = 0.003), but such correlation was not observed between size of CLNM and preablation sTg (ρ = 0.264, *p* = 0.083).

**Table 2:** Univariate analysis of clinicopathologic risk factors for detectable postablation stimulated thyroglobulin level (>0.5 μg/mL)

Characteristic	Detectable postablation sTg	Undetectable postablation sTg	*p*-value
	(*n* = 34)	(*n* = 55)	
Age at operation (years)	43 (21–78)	50 (17–81)	0.065
Sex			0.881
Male	9 (26.5)	13 (23.6)	
Female	25 (73.5)	42 (76.4)	
Clinical presentation			0.063
Incidental	5 (14.7)	24 (43.6)	
Symptomatic	29 (85.3)	31 (56.4)	
Tumor characteristics			
Tumor size (cm)	2.0 (0.3–6.0)	1.5 (0.5–4.0)	**0.018**
Multifocality	16 (47.1)	15 (27.3)	0.125
Capsular invasion	11 (32.4)	10 (18.2)	0.390
Extrathyroidal extension	15 (44.1)	9 (16.4)	0.445
Coexisting thyroiditis	6 (17.6)	13 (23.6)	0.299
Stage of PTC byTNM			0.217
Stage I/II	19 (55.9)	23 (41.8)	
Stage III/IV	15 (44.1)	32 (58.2)	
No. of CLNs retrieved	6 (3–21)	6 (4–18)	0.814
No. of metastatic CLNs excised	2 (0–18)	1 (0–9)	**0.013**
Metastatic CLN ratio (%)^b^	66.7 (0–100)	20 (0–100)	**0.003**
Metastatic CLN (pN1a)	20 (58.8)	29 (52.7)	0.114
Size of CLN metastasis (mm)	4.8 (0.3–6.9)	1.6 (0.1–6.1)	**<0.001**
Type of CLN metastasis			**0.002**
pN0	7 (20.6)	33 (60.0)	
pN1mic	4 (11.8)	16 (29.1)	
pN1mac	23 (67.6)	6 (10.9)	

Boldface signifies *p*-value <0.05 or statistically significant

*sTg* stimulated thyroglobulin, *PTC* papillary thyroid carcinoma, *TNM* 6th edition of the tumor, node and metastasis staging system, *CLN* central lymph node, *pN0* no central lymph node metastasis, *pN1mic* micrometastasis (size ≤ 2 mm), *pN1mac* macrometastasis (size > 2 mm)

^a^Continuous variables are expressed as median (range); categorical variables are expressed as *n* (%)

^b^Metastatic CLN ratio = (no. of metastatic CLNs/no. of CLNs retrieved) × 100

Table 3 shows the multivariate analysis of clinicopathologic risk factors for postablative DsTg 9 months after surgery. Variables that were significant in the univariate analysis were entered into the multivariate analysis. The number of metastatic CLNs was not entered because it significantly correlated with the CLN ratio (ρ = 0.896, *p* < 0.001), and the CLN ratio was shown to be a more significant factor.^21^,^22^ Because the size and type of CLNM were similar covariates, they were entered separately into the multivariate analysis with CLN ratio and tumor size. In the multivariate analysis, after adjusting for CLN ratio and tumor size, size of CLNM (expressed as a continuous variable) (odds ratio 1.56, 95 % confidence interval 1.09–2.24, *p* = 0.015) was the only independent factor for postablation DsTg. Similarly, when the type of CLNM was entered in the multivariate analysis with CLN ratio and tumor size, relative to pN0 and pN1mic, pN1mac was the only independent risk factor for postablation DsTg (odds ratio 1.76, 95 % confidence interval 1.22–27.70, *p* = 0.027).

**Table 3:** Multivariate analysis of clinicopathologic risk factors for postablative detectable stimulated thyroglobulin 9 months after surgery

Covariate	β Coefficient	Odds ratio (95 % confidence interval)	*p*-value
Metastatic CLN ratio^a^	0.014	1.014 (0.990–1.039)	0.251
Primary tumor size	0.389	1.476 (0.848–2.568)	0.168
Type of CLN metastasis^b^			
pN0		1	
pN1mic	0.949	2.583 (0.351–19.000)	0.351
pN1mac	1.760	5.814 (1.221–27.697)	**0.027**

Boldface signifies *p*-value <0.05 or statistically significant

*CLN* central lymph node, *pN0* no CLN metastasis, *pN1mic* micrometastasis (size ≤ 2 mm), *pN1mac* macrometastasis (size > 2 mm)

^a^Metastatic CLN ratio = (no. of metastatic CLNs/no. of CLNs retrieved) × 100

^b^When size of CLN metastasis was entered instead, the odds ratio became 1.564 (95 % confidence interval 1.092–2.241), *p* = 0.015

## Discussion

Previous studies have found that size of LNM in the lateral compartment is a significant risk factor for LRR, distant metastasis and poor overall survival.^8^–^11^,^14^ Sugitani et al. reported that for patients older than 50 years, LNM ≥3 cm was an independent factor for cancer-specific survival from PTC.^10^ Similarly, it was observed that those with clinically apparent or palpable LNM were at significantly higher risk of developing LRR and distant metastases than those with clinically inapparent or nonpalpable LNM.^8^–^13^ In fact, this was the rationale for performing therapeutic selective neck dissection in the lateral compartment and not prophylactic selective neck dissection in patients with PTC.^13^ However, in comparison to lateral selective neck dissection, pCND is associated with lower surgical morbidity and can be performed within the same incision as the thyroidectomy.^23^ In addition, it provides more information regarding the nodal status as required by the TNM and may lower LRRs.^23^ Several studies confirmed that those who underwent pCND had a significantly lower postsurgical sTg than those without pCND. This perhaps suggests that pCND was able to provide a more oncologically complete resection than thyroidectomy alone by excising micrometastases harbored in the central compartment.^4^–^6^ This hypothesis was supported by the fact that over 50 % of CLNs resected harbored metastases.^4^–^7^ However, questions remain regarding the size of these metastases and their importance to patient outcomes. Given that the size of LNM in the lateral compartment is a prognostic factor, we hypothesized that the size of CLNM in pCND would also be a prognostic factor, although this has not yet been demonstrated.

To ensure that our study only evaluated CLNs taken out of pCND, 5 patients with CLNM ≥1 cm were excluded. In our analysis, we found that even when the size of CLNM was limited to <1 cm, it was an independent factor for postablation DsTg 9 months after surgery. There was a significant direct correlation between the size of CLNM and postablation sTg level (ρ = 0.428, *p* = 0.003), although not between size of CLNM and preablation sTg (ρ = 0.264, *p* = 0.083). When the size of CLNM was correlated with other clinicopathologic characteristics, we observed that the size of CLNM was inversely correlated with age (i.e., younger patients had larger-sized CLNM), but this was directly correlated with greater number of CLNs, metastatic CLNs and higher metastatic CLN ratio. This latter association implied that larger-sized CLNM meant more extensive nodal involvement, at least within the central compartment. This would account for the higher rate of DsTg 9 months after surgery in the pN1mac group. In the multivariate analysis, after adjusting for tumor size and CLN ratio, patients with pN1mac were almost 6 times more likely to have postablation DsTg than those with pN0. Despite the small number of patients within each group, it is worth noting that the odds ratio for pN1mic did not reach statistical significance; this may imply that pN1mic did not have the same significance level as pN1mac on postablation DsTg.

However, because the size of CLNM is a histopathologic finding, its clinical application could only be limited to the postsurgical management. Nevertheless, one possible application would be to use it as a factor for deciding on the dose of RAI ablation. Because most patients with either pN1mic or pN1mac would receive at least one ablative dose, it would be rational to have those with pN1mac receiving a higher dose of ablation (instead of the standard dose of 3 GBq) than those with pN1mic or pN0 when RAI ablation is indicated. In our study, it was interesting to observe that up to 42.5 % of patients with either pN0 or pN1mic became athyroglobulinemic after 3 GBq of RAI ablation, whereas only 20.7 % in the pN1mac group became athyroglobulinemic. This means that the latter group might perhaps benefit from a higher dose of RAI ablation if athyroglobulinemia is the aim. If future studies could confirm the size of CLNM as an independent factor for outcomes, reporting the size of CLNM in the histopathology reports could become important.

Despite these findings, we acknowledge shortcomings with our study, including the relatively small number of patients within each group and potential biases in the selection for pCND. As a measure of outcome, some would argue that a sTg of 0.5 μg/L might be set too low and a level taken at 9 months after surgery might be too early. However, as shown previously, a sTg of <0.5 μg/L at 1 year had a >98 % likelihood of identifying patients completely free of disease at follow-up, and a sTg level taken at 1 year after surgery was as good as a level taken 2–3 years after surgery.^24^,^25^ Therefore, we believe sTg <0.5 μg/L at 9 months after surgery is a reasonable surrogate for future recurrences. Although not statistically significant, it is worth noting that of the 3 LRR, all belonged to the pN1mac group and had postablation DsTg with a median sTg level of 87.0 (range 9.7–103.0) μg/L. In contrast, none of the patients in pN0 or pN1mic developed recurrences.

In conclusion, pN1mic accounted for 20 (22.5 %) of 89 patients undergoing pCND. Larger-sized CLNM was significantly associated with younger age, greater number of CLN retrieved, greater number of metastatic CLN excised, higher metastatic CLN ratio and higher postablation sTg level. Tumor size, metastatic CLN ratio and size of CLNM were significantly associated with postablation DsTg, but only size of CLNM turned out to be an independent factor for postablation DsTg. Patients with pN1mac were almost 6 times more likely to have postablation DsTg than those with pN0 or pN1mic.
